# Recent advances in the characterization of genetically defined neurons that regulate internal‐state‐dependent taste modification in mice

**DOI:** 10.14814/phy2.70106

**Published:** 2024-11-10

**Authors:** Ken‐ichiro Nakajima

**Affiliations:** ^1^ Laboratory of Alimentary Neuroscience, Graduate School of Bioagricultural Sciences Nagoya University Chikusa Japan

**Keywords:** brain, hunger, internal state, sodium appetite, taste

## Abstract

The gustatory system plays an important role in evaluating food quality in animals and humans. While some tastes are intrinsically appetitive, such as sweet, which is elicited from high‐calorie nutrients, the other tastes, such as sour and bitter, are aversive and elicited by toxic substances. In mice, taste signals are relayed by multiple regions of the brain, including the nucleus of the solitary tract, and the parabrachial nucleus (PBN) of the pons, before reaching the gustatory cortex via the gustatory thalamus. Recent advances in taste research using mice expressing Cre recombinase in specific neuronal populations, together with chemogenetic/optogenetic tools, have enabled us to identify genetically defined neurons involved in taste transduction pathways across several areas of the brain. While gustatory pathways play a fundamental role in taste transduction, taste preferences are not always stable, but rather vary depending on internal states. This review summarizes recent progress in research on neural circuits that modify the taste information depending on internal states in mice.

## 1 BASIC TASTE TRANSDUCTION MECHANISM IN THE BRAIN

Humans and rodents possess five basic taste modalities: sweet, umami, bitter, sour, and salty. The corresponding taste substances are detected by taste buds with onion‐shaped structures that comprise cells expressing distinct types of taste receptors on the tongue. Taste receptors are mainly divided into G‐protein‐coupled receptors (GPCRs) and ion channels. Among GPCR‐type taste receptors, T1R2 + T1R3 and T1R1 + T1R3 heterodimers detect sweet and umami (L‐amino acids) substances, respectively (Zhao et al., 2003). These receptors are fundamentally important for recognizing caloric sources, wherein T1R3 is essential for sweet and umami taste sensing. In contrast, T2R family receptors are responsible for bitter taste sensing (Mueller et al., 2005). Otop1, a proton‐sensitive ion channel, detects sour substances (Teng et al., 2019; Zhang et al., 2019). By contrast, the detection of saltiness is mediated by several molecular mechanisms. While the taste of a low concentration of sodium (<100 mM) is mediated by ENaC as an appetitive taste, a high concentration of sodium (>300 mM) is mediated by bitter taste cells as an aversive taste (Chandrashekar et al., 2010; Oka et al., 2013). Furthermore, each taste receptor‐expressing cell is specialized for specific taste transduction to the brain via corresponding taste nerves.

Information on taste is sent to the brainstem via the geniculate ganglion and the petrosal portion of the jugular/nodose/petrosal ganglion complex to the rostral nucleus of the solitary tract(rNTS). In rodents, there is a relay from rNTS to the parabrachial nuclei (PBN), which in turn project the information to the ventral posteromedial thalamic nucleus (VPMpc). Eventually, the taste information is received at the primary gustatory cortex, which is a part of the insular cortex (InsCtx), and at the secondary gustatory cortex, also known as the orbitofrontal cortex (OFC) (Fu et al., 2021).

## 2 GENETICALLY DEFINED GUSTATORY NEURONS

Recent advances in taste research using mouse genetics and adeno‐associated virus (AAV) encoding chemogenetic/optogenetic molecular tools in a Cre‐dependent fashion have enabled us to identify genetically defined neurons involved in taste transduction pathways across multiple brain areas. This section provides a summary of recent findings related to the brainstem.

### 2.1 rNTS

In the rNTS, fiber photometry experiments with the fluorescent calcium sensor, GCaMP, have shown that prodynorphin‐expressing neurons (Pdyn neurons) are specifically responsive to sour taste but not to other tastes. While the mice whose Pdyn neurons in the rNTS were genetically ablated maintained high lick numbers toward a sour solution that the control mice usually rejected, optogenetic activation of these neurons evokes aversion. In a three‐port apparatus, mice were provided access to the middle port to lick various solutions (water, bitter solution, or sour solution). They were trained to go to the left (bitter solution or water) or to the right port (sour solution) to report the taste identity. While control mice reported correctly by going to the left port after licking water or bitter solution, Pdyn‐neuron‐activated mice went to the right port after licking water to report sour taste (Zhang et al., 2019). These results suggest that Pdyn neurons in the rNTS are critical for sour taste transduction. In contrast, a similar approach to Pdyn neurons has revealed that somatostatin‐expressing (SST) neurons and calbindin 2‐expressing (Calb2) neurons are critical for sweet and bitter taste transduction in rNTS, respectively (Jin et al., 2021). Collectively, these results suggest the presence of distinct neuronal populations in rNTS that correspond to different taste modalities.

### 2.2 PBN

The PBN, which receives various inputs such as malaise, pain, and energy homeostasis, is known to integrate this information (Palmiter, 2018). Among the various nuclei comprising the PBN, the waist area of the PBN is the region that contains taste‐responsive neurons.

SatB2, a transcription factor, is selectively expressed in the waist area of the PBN. Genetic ablation of SatB2‐expressing neurons impairs sweet taste licking without affecting the licking profiles of the other tastes. In vivo calcium imaging using a miniaturized microscope indicated a selective response to sweet taste solution in SatB2‐expressing neurons (Fu, Iwai, Kondoh, et al., 2019). These results indicate that SatB2‐expressing neurons play a critical role in sweet taste sensing in the PBN. By contrast, the other study has reported that SatB2‐expressing neurons respond to multiple tastes in addition to sweet taste (Jarvie et al., 2021). As shown in this case, labeled line theory (single taste modality is conveyed via a single neural population) and across fiber theory (single taste modality is conveyed via multiple neural populations) in gustatory coding are still under discussion in the field of taste research (Staszko et al., 2020). Although the reason why this discrepancy exists is unclear, the difference in mouse strains used (BAC transgenic versus knock‐in SatB2‐Cre mice) or the differences in the injection site of AAV encoding GCaMP, may influence these results. While SatB2‐expressing neurons project to multiple brain areas, the optogenetic place preference experiments showed that mice preferred a light‐on chamber only when VPMpc (the gustatory thalamus)‐projecting SatB2‐expressing neurons are photostimulated (Fu, Iwai, Kondoh, et al., 2019). These results suggest that the gustatory thalamus‐projecting SatB2‐expressing neurons (but not all SatB2‐expressing neurons in the PBN) transmit sweet appetitive signals to the higher brain area. Interestingly, neuronal silencing experiments of calcitonin gene‐related peptide (CGRP)‐expressing neurons in the PBN have shown an increase in licking in response to bitter or sour tastes but not sweet taste (Jarvie et al., 2021). Furthermore, the double inhibition of CGRP neurons plus SatB2 neurons enhances lick numbers toward bitter and sour taste solutions compared to the single inhibition of CGRP neurons. These results suggest that CGRP neurons (and parts of SatB2 neurons) transmit aversive taste information in the brain. The fact that parts of the CGRP neurons are known to project the gustatory thalamus (Chen et al., 2018) indicates that at least the subpopulation of CGRP neurons is important for aversive taste transduction. Since SatB2 neurons and CGRP neurons do not overlap in the PBN, aversive and appetitive tastes may be processed differently in the PBN.

## 3 NEURONAL CIRCUITS THAT MODIFY TASTE INFORMATION IN A STATE‐DEPENDENT FASHION

Although the number of genetically defined neurons has been reported to increase especially in the brainstem, and their roles in basic gustatory pathways have been revealed, taste preferences are not always stable, but rather vary depending on internal states. In this section, recent findings on the central mechanism for taste modulation corresponding to changes in internal states are presented.

### 3.1 Hunger‐induced taste modification

As indicated by the proverb “hunger is the best sauce,” hunger has been known to elevate food palatability empirically for a long time. Interestingly, sucrose preference increases not only in humans but also in various species such as fruit flies and mice. A recent study revealed that physiological hunger affects preferences for appetitive as well as aversive tastes in a mouse model (Fu, Iwai, Narukawa, et al., 2019).

In the brain, Agouti‐related peptide (AgRP)‐expressing neurons localized in the arcuate nucleus of the hypothalamus (ARC) play a pivotal role in feeding to maintain energy homeostasis (Lowell, 2019). In a brief‐access taste test, artificial hunger mimicked by the optogenetic activation of AgRP neurons elevated sweet taste preference. In contrast, more tolerance of bitter or sour taste was observed after the optogenetic activation of AgRP neurons. By contrast, the chemogenetic inhibition of AgRP neurons suppressed changes in taste preference after overnight fasting. These results suggest that AgRP neurons modulate taste preferences in mice. An electrophysiological study (Li et al., 2013) found that the lateral hypothalamic area (LHA) that is close to the ARC contains two distinct neural populations responding to appetitive and aversive tastes. While AgRP neurons project to multiple brain areas such as the intra‐hypothalamus, the central amygdala, and the brain stem, only LHA‐projecting AgRP neurons change both sweet and bitter taste preferences (Fu, Iwai, Narukawa, et al., 2019). Since AgRP neurons are inhibitory neurons, the chemogenetic interrogation of LHA neurons was performed using Vglut2‐Cre mice whose excitatory neurons selectively expressed Cre and Vgat‐Cre mice whose inhibitory neurons selectively expressed Cre. As a result, the chemogenetic inhibition of glutamatergic LHA neurons, but not GABAergic LHA neurons recapitulated the hunger‐induced taste modification (Fu, Iwai, Narukawa, et al., 2019). Two distinct neuronal pathways from the excitatory LHA neurons to the lateral septum (LS) or to the lateral habenula (LHb) were found to contribute to the modulation of appetitive and aversive taste, respectively (Figure). Anatomical retrograde tracing from the LHA suggested that LS‐projecting LHA neurons rarely overlap with LHb‐projecting LHA neurons.

Since the LS and the LHb are part of the mesolimbic reward system, the LHA may modulate the reward value of the taste. The evidence suggests that the LS acts as a hub of diverse information inputs including anxiety, social aggression, fear, and feeding, and modulates the emotion and motivation (Wirtshafter & Wilson, 2021). Thus, hunger enhances the palatability of sweet taste via the LS. However, further study into the characterization of the downstream network is required.

The LHb is known to be activated in response to diverse aversive stimuli, including pain and bitter taste (Groos & Helmchen, 2024). Since starving animals need to obtain calories, even from decaying foods with bitter or sour taste profiles, a reduction in the neural activity of the LHb during hunger can lead to a greater tolerance of aversive tastes.

### 3.2 Sodium‐depletion induced taste modification

While solutions high in sodium (>300 mM) elicit aversive taste responses, solutions low in sodium (<100 mM) elicit appetitive taste in mice under normal conditions (Zhang et al., 2023). Interestingly, mice prefer high‐sodium solutions under conditions of sodium depletion. This phenomenon is known as “sodium appetite” (Geerling & Loewy, 2008). Recent evidence has shown that sodium appetite is induced by two distinct neuronal populations that elevate the hedonic value of sodium and reduce aversion to high salt concentrations, respectively.

A recent study found that a subset of excitatory Pdyn neurons in the prelocus coeruleus (pre‐LC) in the hindbrain serve as a critical neural substrate for sodium intake behavior (Lee et al., 2019). The optogenetic activation of Pdyn neurons in pre‐LC resulted in increased sodium consumption. By contrast, the optogenetic inhibition of the same neuronal population suppressed high salt preference under conditions of sodium depletion. These results suggest that Pdyn neurons in pre‐LC play a key role in enhancing sodium appetite.

Another study found that prostaglandin E2 receptor (Ptger3) expressing neurons in the subfornical organ (SFO) contribute to the development of tolerance to high salt solutions observed in sodium‐depleted mice (Zhang et al., 2023). The optogenetic activation of Ptger3 neurons in SFO was found to result in thirsty mice with a tendency to lick solutions with a high concentration of sodium that they usually reject. Since the maintenance of sodium homeostasis is vital for animals, these results suggest that sodium depletion leads to an elevation of sodium preference mediated by Pdyn neurons in pre‐LC as well as to a tolerance of salt taste mediated by Ptger3 neurons in SFO to cooperatively maintain sodium homeostasis.

**FIGURE 1:**
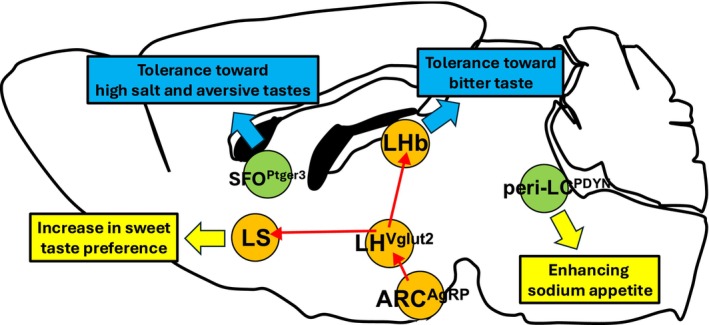
Key genetically defined neurons for internal state‐dependent taste change in mice. Orange circles indicate key sites for hunger‐induced taste modification. Green circles indicate key sites for taste modification during sodium appetite. ARC, arcuate nucleus of the hypothalamus; LH, lateral hypothalamus; LHb, lateral habenula; LS, lateral septum; SFO, subfornical organ; peri‐LC, peri locus ceruleus region.

Although tolerance to aversive tastes has been observed under conditions of energy deprivation and sodium depletion, the neural mechanisms of these two phenomena have been found to differ. While no direct connection has been observed between AgRP neurons in the ARC and Ptger3 neurons in the SFO, their downstream neurons in the development of tolerance toward aversive tastes may be common. By contrast, the neural mechanism that increases preference toward appetitive tastes from caloric sources (ARC➔LH➔LS) is distinct from that for an increased preference for sodium (pre‐LC) (Figure 1).

## 4 FUTURE PERSPECTIVE

Physiological changes have an important effect on taste preferences, such as in the case of fasting and sodium depletion. Changes in their taste preferences help animals maintain physical homeostasis through an adjustment of their dietary preferences and consumption behaviors. However, the question remains as to whether these changes are only observed in terms of physiological changes.

Several studies in humans have found that mental stress affects food preference and the degree of consumption of palatable foods (Choi, 2020; Rutters et al., 2009). Since many factors, including the type of stress, its severity, and individual differences, determine the stress response, mechanistic insights into stress‐induced taste modification remain a key outstanding task of research in this field.

Another crucial topic concerns the mechanisms by which pathophysiological conditions affect taste preferences. Hunger or sodium depletion has been observed to temporally alter taste preferences, reverting to their normal state after homeostasis is recovered. In contrast, while metabolic diseases, such as obesity and diabetes, often lead to a heightened preference for sugar (Hardikar et al., 2017; Ninomiya et al., 1995), their mechanism remains unclear. However, since altered preferences tend to worsen health conditions in patients, understanding the mechanism underlying these changes is likely to hold significant clinical relevance.

## CONFLICT OF INTEREST STATEMENT

The author declares no conflicts of interests.

## ETHICS STATEMEMT

None.
